# Cardiac reserve by 6-minute walk stress echocardiography in systemic sclerosis

**DOI:** 10.1136/openhrt-2020-001559

**Published:** 2021-02-19

**Authors:** Miharu Arase, Kenya Kusunose, Sae Morita, Natsumi Yamaguchi, Yukina Hirata, Susumu Nishio, Yuichiro Okushi, Takayuki Ise, Takeshi Tobiume, Koji Yamaguchi, Daiju Fukuda, Shusuke Yagi, Hirotsugu Yamada, Takeshi Soeki, Tetsuzo Wakatsuki, Masataka Sata

**Affiliations:** 1 Department of Emergency and Critical Care Medicine, Tokushima University Hospital, Tokushima, Japan; 2 Department of Cardiovascular Medicine, Tokushima University Hospital, Tokushima, Japan; 3 Ultrasound Examination Center, Tokushima University Hospital, Tokushima, Japan; 4 Department of Cardio-Diabetes Medicine, Tokushima University Graduate School of Medicine, Tokushima, Japan; 5 Department of Community Medicine for Cardiology, Tokushima University Graduate School of Medicine, Tokushima, Japan

**Keywords:** echocardiography, cardiac imaging techniques, diagnostic imaging

## Abstract

**Objectives:**

There is a high prevalence of left ventricular diastolic dysfunction (LVDD) in systemic sclerosis (SSc) which is associated with high mortality. Thus, early detection of LVDD could be important in management of SSc. We hypothesised that exercise echocardiography in SSc patients with normal resting haemodynamics may expose early phase LVDD, which could affect its prognosis, defined as cardiovascular death and unplanned hospitalisation for heart failure.

**Methods:**

Between January 2014 and December 2018, we prospectively enrolled 140 patients with SSc who underwent 6-minute walk (6MW) stress echocardiographic studies with normal range of estimated mean pulmonary arterial pressure (mPAP) (<25 mm Hg) and mean pulmonary artery wedge pressure (mPAWP) (<15 mm Hg) at rest. We used ΔmPAP/Δcardiac output (CO) to assess pulmonary vascular reserve and ΔmPAWP/ΔCO to assess LV cardiac reserve between resting and post-6MW.

**Results:**

During a median period of 3.6 years (IQR 2.0–5.1 years), 25 patients (18%) reached the composite outcome. Both ΔmPAP/ΔCO and ΔmPAWP/ΔCO in patients with events were significantly greater than in those without events (8.9±3.8 mm Hg/L/min vs 3.0±1.7 mm Hg/L/min; p=0.002, and 2.2±0.9 mm Hg/L/min vs 0.9±0.5 mm Hg/L/min; p<0.001, respectively). Patients with both impaired LV cardiac reserve (ΔmPAWP/ΔCO>1.4 mm Hg/L/min) and impaired pulmonary vascular reserve (ΔmPAP/ΔCO>3.0 mm Hg/L/min) had worse outcomes compared with those without these abnormalities (p<0.001).

**Conclusion:**

The 6MW stress echocardiography revealed impaired LV cardiac reserve in SSc patients with normal resting haemodynamics. Furthermore, LV cardiac reserve independently associates with clinical worsening in SSc, providing incremental prognostic utility, in addition to pulmonary vascular parameters.

**Key questions**

**What is already known on this subject?**

- The myocardial involvement of systemic sclerosis (SSc) clinically causes cardiac dysfunction, especially left ventricular diastolic dysfunction (LVDD). Several studies have shown that LVDD in SSc is highly prevalent and is associated with increased risk of clinical worsening. In addition, LVDD may occur before clinical manifestation.

**What does this study add?**

- ΔmPAP/ΔCO and ΔmPAWP/ΔCO in the event group were significantly increased compared with the non-event group.
- Impaired LV cardiac reserve and pulmonary vascular reserve were independent predictors of clinical worsening in SSc.

**How might this impact on clinical practice?**

- Exercise echocardiography may reveal an early stage of LVDD, despite showing normal resting filling pressure.
- These results indicate that 6-minute stress echocardiographic study may predict a high-risk group of patients with SSc.

## Introduction

Systemic sclerosis (SSc) is an autoimmune connective tissue disease characterised by fibrosis of the skin and internal organs, and vasculopathy.^1^ It is well known that SSc complications are secondary to pulmonary arterial hypertension (PAH), interstitial lung disease and renal disease.^2 3^ Moreover, primary cardiac involvement in SSc, such as myocardial damage, fibrosis of the conduction system, pericardial and valvular disease, is a common complication of SSc.^2^ The general pathogenetic process at the myocardial level leads to focal recurrent ischaemia and immunoinflammatory damage, resulting in myocardial fibrosis.^4^ This myocardial involvement clinically causes cardiac dysfunction, especially left ventricular diastolic dysfunction (LVDD). Several studies have shown that LVDD in SSc is highly prevalent (23%–62%) and is associated with increased risk of mortality.^5–7^ In addition, LVDD may occur before clinical manifestation. Thus, early detection of LVDD in patients with SSc is clinically important.

Recently, it has been demonstrated that exercise echocardiography may evaluate an early stage of LVDD, despite showing normal resting filling pressure.^8^ Several stress methods are extensively used in the clinical setting, and among them, a 6-minute walk (6MW) test is a simple, easy, inexpensive and widely used method. In fact, our previous studies showed that measuring Δmean pulmonary artery pressure (mPAP)/Δcardiac output (CO) by 6MW stress echocardiography can be associated with development of pulmonary hypertension (PH); thus, it is effective in assessing right-sided haemodynamic responses in SSc.^9^ In contrast, the balance of mean pulmonary arterial wedge pressure (mPAWP) and cardiac output (CO) is essential for the assessment of early LVDD.^10^ From the viewpoint of the Frank-Starling mechanism, CO increases as LV filling pressure (eg, PAWP) elevates in normal subjects. However, patients with severely impaired diastolic dysfunction are unable to augment their cardiac output effectively even though LV filling pressure is significantly elevated. We hypothesised that SSc patients with normal resting haemodynamics may present an early phase of LVDD measured by mPAWP and CO with exercise echocardiography, and lead to a model to predict long-term outcomes. We sought to (1) evaluate early phase LVDD with a 6MW stress echocardiographic study in our SSc group; and (2) assess the prognosis in those patients using variables of 6MW stress echocardiography.

## Methods

### Data sharing statement

Individual anonymised data supporting the analyses contained in the manuscript will be made available on reasonable written request from researchers whose proposed use of the data for a specific purpose has been approved.

### Patient and public involvement

Patients or the public were not involved in the design, or conduct, reporting or dissemination plans of our research.

### Study population

**Figure 1:**
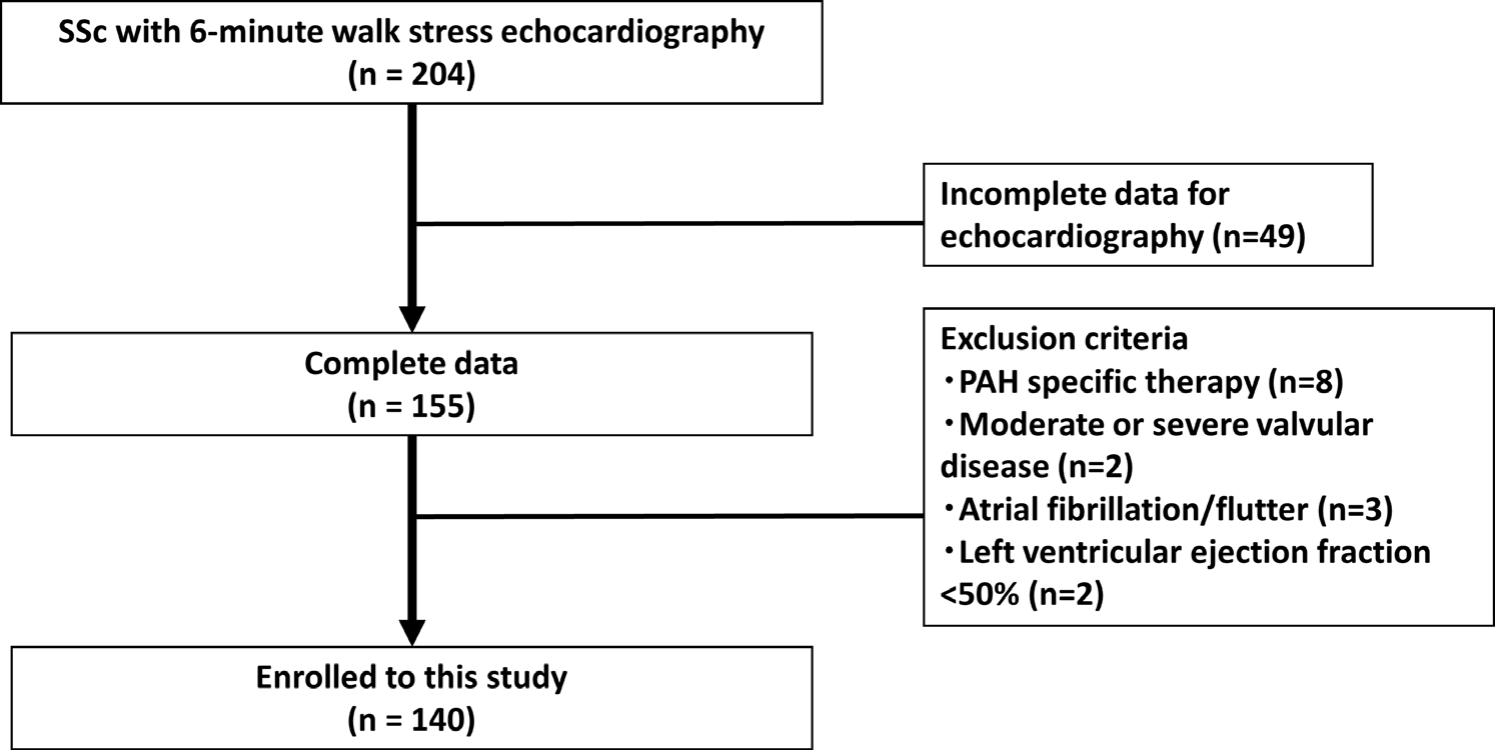
Patient selection. PAH, pulmonary arterial hypertension; SSc, systemic sclerosis.

We prospectively enrolled consecutive patients with SSc who underwent 6MW stress echocardiographic studies and had normal range of estimated mean pulmonary arterial pressure (mPAP) (<25 mm Hg) and normal range of estimated mPAWP (<15 mm Hg) at rest. Patients were referred to our echocardiographic examination centre between January 2014 and December 2018. Definitions of SSc were based on the American College of Rheumatology diagnostic criteria.^11^ Disease duration was defined as time from diagnosis to baseline echocardiography, and the observation period as time from baseline echocardiography to date of death or study end (December 2019). Figure 1 shows a selection of eligible patients and the enrolment process. Forty-nine patients at rest or during stress test were excluded due to incomplete measurable echocardiographic parameters (lack of tricuspid regurgitant velocity; n=26, lack of mitral annular velocity; n=20, and poor image quality; n=3). We also excluded patients treated with PAH-specific therapies at baseline (n=8), with moderate or severe valvular disease (n=2), atrial fibrillation/flutter (n=3), left ventricular ejection fraction <50% (n=2). One hundred forty patients fulfilled all criteria for the final analysis.

### Echocardiographic assessment

Transthoracic echocardiography was performed by experienced sonographers/doctors using a commercially available ultrasound machine (Vivid 9, GE Vingmed, Horten, Norway). Measurements and recordings were obtained according to the American Society of Echocardiography recommendations.^12^ Systolic PAP was measured from the maximal continuous-wave Doppler velocity of the tricuspid regurgitant jet using systolic trans-tricuspid pressure gradient calculated by the modified Bernoulli equation. Right atrial pressure was estimated from the inferior vena cava diameter and collapsibility.^13^ The mPAP was calculated as 0.6×systolic PAP+2.^14^ The mPAWP was calculated as 1.24×(E/e')+1.9 using the Nagueh formula.^15^ Pulmonary vascular resistance (PVR) was calculated as (mPAP‒mPAWP)/CO.

Peak systolic longitudinal strain measurements were obtained from greyscale images recorded in the apical 4-chamber, 2-chamber and long-axis views. The frame rate was maintained at >40 frame/s. All strains were analysed offline using speckle tracking vender-independent software (EchoInsight, Epsilon Imaging, Ann Arbor, Michigan, USA). Global longitudinal strain (GLS) was obtained by averaging all segmental strain values from the apical 4-chamber, 2-chamber and long-axis views. In RV longitudinal strain analysis from the RV focused apical 4-chamber view, the interventricular septum was included in the region-of-interest for speckle-tracking echocardiography, but only the free wall strain values were included and the septal strain values were discarded to avoid LV interaction. GLS and RV longitudinal strain were obtained at rest.

### Six-minute walk stress echocardiography

Online supplemental clip shows the 6MW stress echocardiography. The 6MW tests were performed according to the American Thoracic Society guidelines.^16^ The transcutaneous arterial oxygen saturation was determined by pulse oximetry. As we performed in our previous study,^9^ post-6MW (within 30 s), early diastolic transmitral flow/annular velocity and the peak tricuspid regurgitation jet were obtained immediately. CO was also obtained from electric cardiometry (Aesculon Electrical Velocimetry, Osypka Medical GmbH, Berlin, Germany) at the same time in order to check the changes of CO immediately after the 6MW test.^17^ Because it was difficult to measure many indices immediately after the 6MW test, we used electric cardiometry to obtain CO. We calculated the slope of mPAWP/CO and mPAP/CO in individual patients (ΔmPAWP/ΔCO and ΔmPAP/ΔCO).

10.1136/openhrt-2020-001559.supp1 Supplementary video

In 14 patients, exercise right heart catheterisation was performed using a Swan-Ganz catheter to assess the actual haemodynamics. Pressure measurements were obtained at rest and during supine bicycle ergometery. The workload was increased at 25 W increments every 3 min. Average peak workload was 50 W in our cohort. The following haemodynamic parameters were recorded at the peak workload: PAWP, mPAP and CO. Thermodilution CO were analysed after averaging the sum of three measurements at rest and during exercise.

### Clinical outcomes

All patients were followed up at our hospital according to the research protocol (follow-up every 3 months). The composite outcome was time to clinical worsening, which was determined on the basis of time from baseline to the first occurrence of any of the following: cardiovascular death or unplanned hospitalisation for heart failure. Unplanned hospitalisation for heart failure was defined by an independent adjudicator as an overnight admission for heart failure (excluding emergency department only stays) which was not for an elective purpose or procedure. The duration of follow-up was begun at the time of the initial stress echocardiogram and ended in December 2019. The 6MW stress echocardiographic data were blinded to physicians at follow-up clinic after study initiation.

### Statistical analysis

Data are presented as mean±SD. Statistical significance of differences between the groups was assessed using the Student’s t-test for data with normal distribution, and the Mann-Whitney U test was used for data that were not normally distributed. For categorical variables, the Fisher’s exact test was used. The association of clinical variables with outcome was identified by Cox proportional hazards models in univariate and multivariate analyses. A HR with a 95% CI was calculated for each variable. The scaled Schoenfeld residuals for each independent variable were plotted against time to assess the assumption of proportional hazards; these correlations were found to be non-significant. Receiver operating characteristic (ROC) curves were generated to determine optimal cut-off values of continuous variables. The best cut-off value was defined as the upper limit of the CI of the Youden index. To assess prognostic value, reference values of ΔmPAP/ΔCO (3.0 mm Hg/L/min based on our previous paper)^9^ and ΔmPAWP/ΔCO (1.4 mm Hg/L/min based on ROC analysis) were used to divide patients into two groups for Kaplan-Meier analysis, with event-free survival compared using a two-sided log-rank test. Statistical analysis was performed using standard statistical software packages (SPSS software V.21.0, SPSS; MedCalc software V.17; Mariakerke, Belgium). Statistical significance was defined by p<0.05.

## Results

### Patient characteristics

Baseline characteristics of the study group are presented in table 1. The study population consisted of 140 patients (61±12 years; 10% male) who underwent 6MW stress echocardiography. Those patients were divided into two groups with and without events. Both study groups had short disease duration (1.5 and 1.7 months, respectively). Also, complications such as respiratory dysfunction or abnormal haemodynamics at baseline (eg, ischaemic heart disease, heart failure, atrial fibrillation, hypertension) were not present in either group, thus no medication treating common complications of SSc was administrated when the 6MW test was performed. Heart rate and blood pressure at baseline were well-controlled in this cohort (heart rate: 68±12 beats/min and systolic blood pressure: 123±20 mm Hg). Post-6MW, heart rate and blood pressure were increased, and percutaneous oxygen saturation was slightly decreased. The correlation between invasive and non-invasive (electric cardiometry and echocardiography) values is shown in online supplemental figure 1. The invasive data are shown in online supplemental table 1. In this small cohort, there was a good correlation between invasive and non-invasive of ΔmPAWP/ΔCO (r=0.78; p<0.001). The intraobserver variability for the measurement of ΔmPAWP/ΔCO was 6.5%±2.8%; interobserver variability was 8.2%±4.6%. Echocardiographic variables are shown in table 2. Post-6MW, CO, mPAP and mPAWP were increased. The average ΔPAWP/ΔCO was 1.1±0.6 mm Hg/L/min and the average ΔmPAP/ΔCO was 4.1±2.5 mm Hg/L/min. There was no difference in PVR between event (+) and event (-) groups in the resting and exercise states.

10.1136/openhrt-2020-001559.supp2 Supplementary data

10.1136/openhrt-2020-001559.supp3 Supplementary data

**Table 1:** Clinical characteristics

	All	Event (+)	Event (-)	P value
Number	140	25	115	–
Age	61±12	62±10	60±12	0.54
Male, %	14 (10)	1 (4)	13 (11)	0.11
Body mass index	22±3	22±3	22±3	0.88
WHO Class I/II/III/IV	27/82/31/0	5/16/4/0	22/66/27/0	0.54
**History**				
Disease duration, months	1.7	1.5	1.7	0.75
**Medication**				
Antihypertensive drugs, %	2 (1)	0 (0)	2 (2)	0.16
Diuretic, %	3 (2)	1 (4)	2 (2)	0.59
Anticoagulants, %	0 (0)	0 (0)	0 (0)	–
**Respiratory function**				
%EFV_1_, %	80±4	82±6	79±4	0.82
%FVC, %	109±13	113±15	107±12	0.34
%DLCO	80±13	81±14	79±11	0.53
**Baseline haemodynamics**				
HR, bpm	68±12	68±10	67±12	0.67
Systolic BP, mm Hg	123±20	122±22	123±19	0.93
Diastolic BP, mm Hg	68±10	68±10	69±10	0.68
SpO_2_, %	98±1	97±2	98±1	0.53
**Post-6-minute walk haemodynamics**				
HR, bpm	90±17	92±18	89±16	0.54
Systolic BP, mm Hg	128±25	129±22	128±25	0.79
Diastolic BP, mm Hg	66±15	70±19	65±14	0.22
SpO_2_, %	96±3	96±2	96±3	0.80
6MW distance, m	432±89	451±94	428±87	0.26

Data are presented as number of patients (percentage) and mean±SD.

BP, blood pressure; %DLCO, diffusing capacity for carbon monoxide; %FEV1, percent forced expiratory volume in 1 s; %FVC, percent forced vital capacity; HR, heart rate; MCTD, mixed connective tissue disease; SpO_2_, percutaneous oxygen saturation; SSc, systemic sclerosis.

**Table 2:** Haemodynamic parameters

	All	Event (+)	Event (-)	P value
**Rest echocardiographic variables**				
LVEDVi, mL/m^2^	49±11	49±14	49±10	0.87
LVESVi, mL/m^2^	17±4	17±6	17±4	0.59
LVEF, %	65±4	66±3	65±4	0.15
LVMi, g/m^2^	75±17	72±19	76±16	0.31
LAVi, mL/m^2^	29±9	29±12	28±9	0.79
e’, cm/s	10±3	11±3	10±3	0.27
E/e'	7±3	8±4	7±3	0.09
RVEDA, cm^2^	14±3	13±3	14±3	0.10
RVESA, cm^2^	8±2	8±2	8±2	0.78
RVFAC, %	44±10	41±12	45±10	0.14
TAPSE, mm	22±4	21±4	22±4	0.50
GLS, %	19±3	18±2	19±3	0.19
RVLS, %	25±6	25±7	25±6	0.87
**Exercise haemodynamics**				
Mean PAP, mm Hg	17±3	18±3	17±3	0.29
Mean PAWP,	11±4	12±4	11±4	0.09
CO, L/min	3.8±1.2	4.1±1.7	3.8±1.1	0.41
PVR, wood unit	1.9±1.1	1.9±0.8	1.9±1.2	0.57
Exercise mean PAP, mm Hg	23±5	25±4	23±5	0.05
Exercise mean PAWP, mm Hg	13±4	14±5	12±4	0.04
Exercise CO, L/min	6.1±2.1	5.6±2.1	6.2±2.1	0.19
Exercise PVR, wood unit	1.9±1.0	2.2±1.1	1.9±0.9	0.17
ΔmPAP/ΔCO, mm Hg/L/min	4.1±2.5	8.9±3.8	3.0±1.7	0.008
ΔmPAWP/ΔCO, mm Hg/L/min	1.1±0.6	2.2±0.9	0.9±0.5	0.004
Δe’, cm/s	0.9±3.1	0.5±3.6	1.0±3.1	0.63

Data are presented as number of patients (percentage) and mean±SD.

CO, cardiac output; e’, early diastolic mitral annular motion; E, early diastolic transmitral flow velocity; LAVi, left atrial volume index; LVEDVi, left ventricular end-diastolic volume index; LVEF, left ventricular ejection fraction; LVESVi, left ventricular end-systolic volume index; LVMi, left ventricular mass index; mPAP, mean pulmonary artery pressure; mPAWP, mean pulmonary artery wedge pressure; PVR, pulmonary vascular resistance; RVEA, right ventricular end-diastolic area; RVESA, right ventricular end-systolic area; RVFAC, right ventricular functional area change; TAPSE, tricuspid annular plane systolic excursion.

### Event-free survival

**Figure 2:**
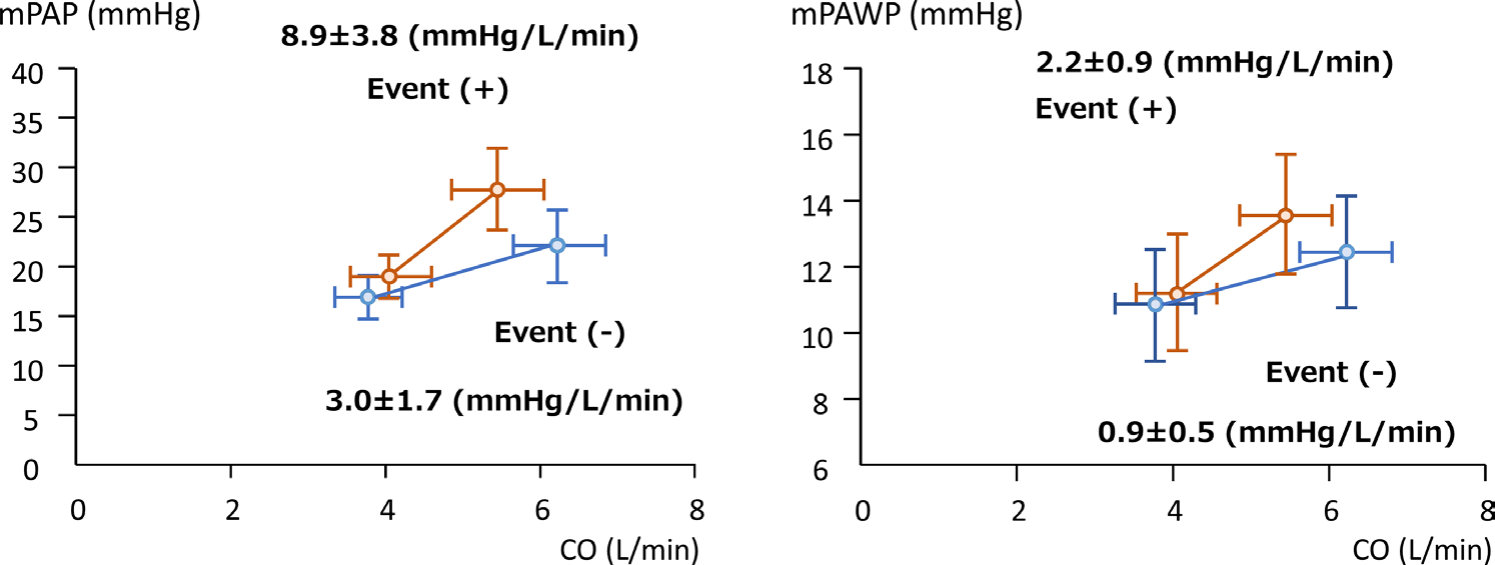
Multipoint for mPAWP-mPAP and cardiac output. (A) Multipoint mPAP-CO plots at baseline and post-6MW. ΔmPAP/ΔCO with events (the red line) is significantly greater than ΔmPAP/ΔCO without events (the blue line) (8.9±3.8 mm Hg/L/min vs 3.0±1.7 mm Hg/L/min; p=0.002). (B) Multipoint mPAWP-CO plots at baseline and post-6MW. ΔmPAWP/ΔCO with events (the red line) is significantly greater than ΔmPAWP/ΔCO without events (the blue line) (2.2±0.9 mm Hg/L/min vs 0.9±0.5 mm Hg/L/min; p<0.001). 6MW, 6-minute walk; CO, cardiac output; mPAP, mean pulmonary artery pressure; mPAWP, mean pulmonary artery wedge pressure.

During a median period of 3.6 years (IQR 2.0–5.1 years), 25 patients (18%) reached the composite outcome (cardiovascular death due to heart failure, n=1; unplanned hospitalisation for heart failure, n=24). HRs of the relevant parameters in univariate models are shown in table 3. At rest parameters, E/e’ (HR 1.09; 95% CI 1.01 to 1.19, p=0.04) and mPAWP (HR 1.07; 95% CI 1.00 to 1.15; p=0.04) were associated with time to clinical worsening. At exercise parameters, the exercise mPAWP (HR 1.08; 95% CI 1.02 to 1.15, p=0.012), ΔmPAP/ΔCO (HR 1.06; 95% CI 1.02 to 1.10, p=0.002) and ΔmPAWP/ΔCO (HR 1.38; 95% CI 1.17 to 1.63, p<0.001) were associated with time to clinical worsening. In addition, ΔCO was also associated with adverse outcomes (HR 0.58; 95% CI 0.38 to 0.87, p=0.009). Figure 2 shows multipoint mPAWP-CO plots and mPAP-CO plots at baseline and post-6MW. The ΔmPAP/ΔCO in patients with events was significantly greater than in patients without events (8.9±3.8 mm Hg/L/min vs 3.0±1.7 mm Hg/L/min; p=0.002). Moreover, the ΔmPAWP/ΔCO in patients with events was also significantly greater than in patients without events (2.2±0.9 mm Hg/L/min vs 0.9±0.5 mm Hg/L/min; p<0.001). Using an ROC curve, we found that the best cut-off value of ΔmPAWP/ΔCO for predicting events was >1.4 mm Hg/L/min with 60% sensitivity and 80% specificity.

**Table 3:** Univariate associations of outcomes

	Univariate model		
	HR	95% CI	P value
Age	1.02	0.98 to 1.05	0.54
Male, %	0.41	0.01 to 9.17	0.25
Disease duration	0.99	0.99 to 1.01	0.75
HR, bpm	1.00	0.97 to 1.04	0.87
Systolic BP, mm Hg	0.99	0.98 to 1.02	0.72
SpO_2_, %	0.93	0.73 to 1.19	0.57
**Rest echocardiographic variables**			
LVEF, %	1.01	0.95 to 1.07	0.86
LAVi, mL/m^2^	1.01	0.97 to 1.05	0.73
E/e'	1.09	1.01 to 1.19	0.04
GLS	0.88	0.71 to 1.10	0.26
RVLS	0.97	0.87 to 1.09	0.67
**Exercise haemodynamics**			
mPAP, mm Hg	1.09	0.95 to 1.26	0.22
mPAWP, mm Hg	1.07	1.00 to 1.15	0.04
CO, L/min	1.14	0.84 to 1.55	0.41
Exercise mPAP, mm Hg	1.06	1.00 to 1.13	0.07
Exercise mPAWP, mm Hg	1.08	1.02 to 1.15	0.012
Exercise CO, L/min	0.84	0.66 to 1.06	0.14
ΔmPAWP	1.27	1.01 to 1.61	0.049
ΔCO	0.58	0.38 to 0.87	0.009
ΔmPAP/ΔCO, mm Hg/L/min	1.06	1.02 to 1.10	0.002
ΔmPAWP/ΔCO, mm Hg/L/min	1.38	1.17 to 1.63	<0.001
Δe’, cm/s	0.95	0.84 to 1.07	0.38

BP, blood pressure; CO, cardiac output; %DLCO, diffusing capacity for carbon monoxide; e’, early diastolic mitral annular motion; E, early diastolic transmitral flow velocity; %FEV1, percent forced expiratory volume in 1 s; %FVC, percent forced vital capacity; HR, heart rate; LAVi, left atrial volume index; LVEDVi, left ventricular end-diastolic volume index; LVEF, left ventricular ejection fraction; LVESVi, left ventricular end-systolic volume index; LVMi, left ventricular mass index; MCTD, mixed connective tissue disease; mPAP, mean pulmonary artery pressure; mPAWP, mean pulmonary artery wedge pressure; PVR, pulmonary vascular resistance; RVEA, right ventricular end-diastolic area; RVESA, right ventricular end-systolic area; RVFAC, right ventricular functional area change; SpO_2_, percutaneous oxygen saturation; SSc, systemic sclerosis; TAPSE, tricuspid annular plane systolic excursion.

**Figure 3:**
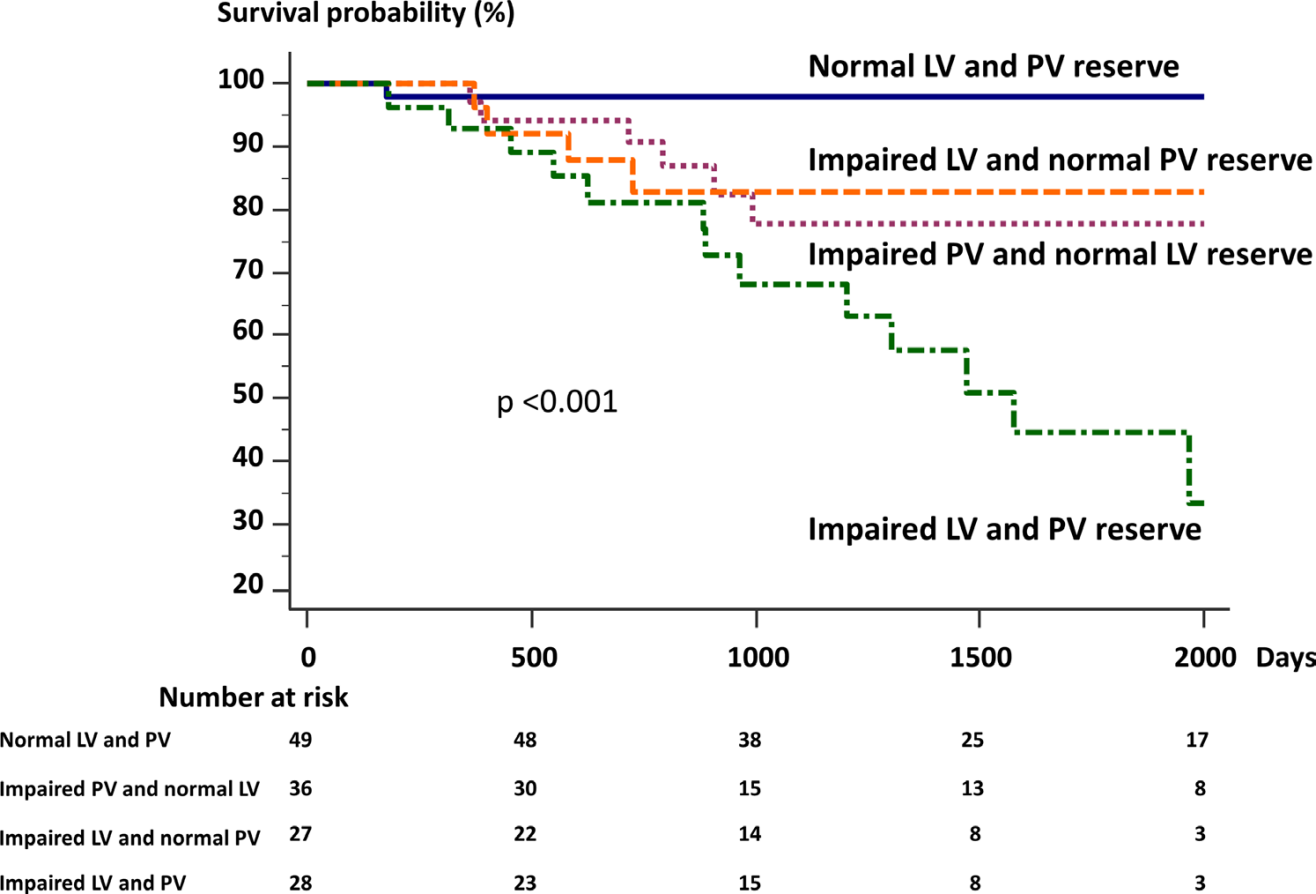
Kaplan-Meier analysis of event-free survival. Patients were stratified according to pulmonary vascular (PV) reserve (ΔmPAP/ΔCO) and left ventricular (LV) cardiac reserve (ΔmPAWP/ΔCO). Patients with abnormal ΔmPAP/ΔCO (>3.0 mm Hg/L/min) or ΔmPAWP/ΔCO (>1.4 mm Hg/L/min) are strongly associated with shorter event-free survival. Moreover, patients with both impaired LV and PV function had worst outcomes.

In the multivariate model, ΔmPAP/ΔCO, ΔmPAWP/ΔCO and their interaction were selected to test the clinical utility of these variables (table 4). Interestingly, ΔmPAP/ΔCO (HR 1.18; 95% CI 1.05 to 1.32, p=0.006), ΔmPAWP/ΔCO (HR 2.12; 95% CI 1.46 to 3.06, p<0.001) and their interaction (HR 0.96; 95% CI 0.93 to 0.99, p=0.016) were significantly associated with time to clinical worsening. Figure 3 illustrates patient time to event stratified according to ΔmPAP/ΔCO and ΔmPAWP/ΔCO to check the influence of this interaction. Patients with impaired pulmonary vascular (PV) reserve (ΔmPAP/ΔCO>3.0 mm Hg/L/min) and impaired LV cardiac reserve (ΔmPAWP/ΔCO>1.4 mm Hg/L/min) had significantly shorter event-free survival than those without these abnormalities.

**Table 4:** Multivariate associations of outcomes

	Multivariate model		
	HR	95% CI	P value
ΔmPAP/ΔCO, mm Hg/L/min	1.18	1.05 to 1.32	0.006
ΔmPAWP/ΔCO, mm Hg/L/min	2.12	1.46 to 3.06	<0.001
ΔmPAP/ΔCO×ΔmPAWP/ΔCO	0.96	0.93 to 0.99	0.016

CO, cardiac output; mPAP, mean pulmonary artery pressure; mPAWP, mean pulmonary artery wedge pressure.;

Table 5 shows the clinical characteristics and haemodynamic variables in categorised groups by LV and PV reserve. There was no difference in haemodynamic parameters at rest among the groups. On the other hand, exercise haemodynamic parameters were significantly different compared with normal LV and PV reserve in the other groups. In univariate Cox proportional hazard analysis, the HRs for each group of LV and PV reserve using the normal LV and PV groups as a reference are shown in table 5. Patients with impaired PV reserve and LV reserve had the highest risk for outcomes among the groups (HR 35.4; 95% CI 4.6 to 271, p<0.05).

**Table 5:** Clinical characteristics in categorised groups by LV and PV reserve

	Norma LV and PV reserve	Impaired PV and normal LV reserve	Impaired LV and normal PV reserve	Impaired LV and PV reserve
Number	49	36	27	28
Age	57±12	61±12	64±10	63±11
Male, %	5 (10)	6 (17)	1 (4)	2 (7)
**Rest echocardiographic variables**				
LVEDVi, mL/m^2^	50±10	50±14	48±11	48±9
LVESVi, mL/m^2^	17±4	17±5	17±4	17±4
LVEF, %	65±4	66±3	64±3	66±4
LVMi, g/m^2^	76±17	76±15	74±20	74±15
LAVi, mL/m^2^	27±8	27±10	32±7	29±11
e’, cm/s	11±3	11±3	10±2	10±3
E/e'	6±2	8±3	8±4	7±3
RVEDA, cm^2^	14±3	15±4	14±3	14±3
RVESA, cm^2^	8±2	8±2	8±2	8±2
RVFAC, %	44±11	46±9	43±10	44±10
GLS, %	19±2	19±4	19±2	19±3
RVLS, %	24±5	26±6	24±7	25±7
**Exercise haemodynamics**				
Mean PAP, mm Hg	17±2	17±4	18±2	18±3
Mean PAWP, mm Hg	10±2	11±5	12±4	11±4
CO, L/min	3.9±1.1	3.9±1.6	3.7±1.0	3.7±0.9
PVR, wood unit	1.9±0.9	1.8±0.9	1.8±0.8	2.4±1.8
Exercise mean PAP, mm Hg	21±4	24±5*	21±3	26±6*
Exercise mean PAWP, mm Hg	11±3	12±5	15±4*	14±5*
Exercise CO, L/min	7.3±2.5	5.5±1.7*	5.8±1.8*	4.9±0.9*
Exercise PVR, wood unit	1.6±0.6	2.4±0.8*	1.3±0.6	2.7±1.4*
ΔmPAP/ΔCO, mm Hg/L/min	1.6±0.7	5.0±2.0*	1.8±0.7	9.5±6.0*
ΔmPAWP/ΔCO, mm Hg/L/min	0.3±0.2	0.7±0.4	1.5±0.5*	3.0±1.7*
**HR for outcomes**	Reference	9.9 (1.2–83.1)*	8.4 (1.1–72.8)*	35.4 (4.6–271)*

*Versus normal LV and PV, p<0.05.

CO, cardiac output; e’, early diastolic mitral annular motion; E, early diastolic transmitral flow velocity; LAVi, left atrial volume index; LVEDVi, left ventricular end-diastolic volume index; LVEF, left ventricular ejection fraction; LVESVi, left ventricular end-systolic volume index; LVMi, left ventricular mass index; mPAP, mean pulmonary artery pressure; mPAWP, mean pulmonary artery wedge pressure; PVR, pulmonary vascular resistance; RVEA, right ventricular end-diastolic area; RVESA, right ventricular end-systolic area; RVFAC, right ventricular functional area change; TAPSE, tricuspid annular plane systolic excursion.

## Discussion

Our study brings several new insights into the understanding of cardiac and pulmonary functions in patients with SSc: (1) The ΔmPAP/ΔCO and ΔmPAWP/ΔCO in the event group was significantly increased compared with that of the non-event group. (2) Impaired LV cardiac reserve and PV reserve were independent predictors of clinical worsening. (3) There was good correlation between echocardiography-based parameters after 6MW testing and catheter-based parameters during ergometer stress. We believe that the reliability of the 6MW stress echocardiography is acceptable in this cohort. Those results indicated that a 6MW stress echocardiographic study may predict a high-risk group in patients with SSc, even within 3 months after diagnosis. The invasive measurements were made during peak exercise load during ergometric testing, a good correlation may be helpful to convince those who are concerned about the reliability of the 6MW echo test, where measurements are performed after the exercise.

### Diastolic dysfunction in sclerosis

Recently, there has been focused discussion on the importance of LVDD in SSc. Previous studies demonstrated that LVDD has a high prevalence and may be an independent predictor of mortality in SSc.^18^ Although diastolic dysfunction is often one of the earliest signs of cardiac impairment in SSc, it is not always manifested in a resting state. Nevertheless, the early stage of LVDD with normal resting haemodynamics can be diagnosed by an exercise stress test. The previous study found that exercise PAWP may be associated with outcome in early stage heart failure.^19^ Moreover, the E/e’ exercise ratio correlated with LV filling pressure (eg, PAWP) and predicted adverse cardiovascular outcomes.^20 21^ Those reports are consistent with our analysis that exercise mPAWP calculated by the E/e’ ratio is associated with adverse outcomes in our SSc group.

The balance of PAWP and CO is key to evaluate LVDD. As long as the Frank-Starling mechanism functions effectively, CO increases after dynamic preload stress; however, it does not apply to patients with severely impaired LV relaxation, which results in only slightly increased CO on significantly elevated PAWP.^10^ It has also been reported that decreased peak CO measured by an exercise stress test is associated with poor prognosis in congestive heart failure.^22^ Those studies are compatible with our result, in which mPAWP/CO was increased after the 6MW test compared with resting, especially in the events group. In addition, among our small cohort, good correlation between the catheter and echocardiography of ΔmPAWP/ΔCO was observed at rest and during exercise. It indicates that the 6MW stress echocardiographic study is useful to detect early LVDD without invasive measurement and to predict outcome in patients with SSc despite normal resting haemodynamics.

### Pulmonary vascular function in sclerosis

PH is a severe complication of SSc, and currently early detection and management of PAH is recommended.^23^ ΔmPAP/ΔCO with 6MW stress echocardiography has been shown to be a good predictor of PH development in connective tissue diseases including SSc.^9^ Our results confirmed that ΔmPAP/ΔCO measured by the 6MW test predicts a worse outcome in patients with SSc. However, the possibility remains that LVDD leads to an elevated left atrial pressure, resulting in PH (WHO Group 2) which affects prognosis. In fact, it has been reported that PH related to SSc can sometimes be associated with occult left-sided diastolic dysfunction.^24^ Also, SSc patients with PH due to early LVDD showed a twofold increased risk of death, compared with isolated patients with SSc-PAH.^25^ Furthermore, LVDD might affect mortality independently in patients with SSc. Thus, the assessment of both PV function and early diastolic dysfunction is necessary to measure the prognosis of patients with SSc. In addition, the RV-PA coupling index may be useful as an early prognostic indicator in early SSc.^26^ It could be an important indicator in this area in the future.

In our analysis, although most of the resting echocardiographic measurements, including GLS and RVLS, did not have predictive values in SSc, the value of 6MW stress echocardiography related to clinical worsening in both LV cardiac reserve (ΔmPAWP/ΔCO) and PV reserve (ΔmPAP/ΔCO). Importantly, CO change was a more prominent factor associated with adverse outcomes in univariate analysis. CO is a key diagnostic parameter and a major prognostic factor in PAH. We should take CO into account when we assess pulmonary artery pressure. In the setting of impaired PV reserve, 6MW stress echocardiography revealed that SSc patients with LVDD had worse prognoses than those without LVDD, consistent with previous studies. Interestingly, in the setting of normal PV reserve, SSc patients with LVDD had worse prognosis than those without LVDD. These results indicate that impaired LV cardiac reserve in SSc, enhanced by 6MW stress echocardiography, can be an independent prognostic factor prior to development of complications, including PH, or treatment, since our cohort was examined at an initial naïve state. Therefore, it is essential to assess LV function as soon as SSc is diagnosed.

### Clinical implications

**Figure 4:**
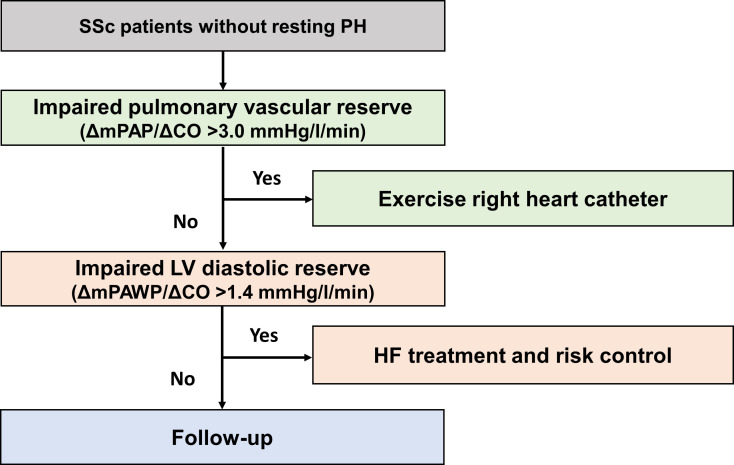
Potential approach to exercise haemodynamics in systemic sclerosis (SSc). We propose a potential approach to identify a high-risk group in SSc. CO, cardiac output; mPAP, mean pulmonary artery pressure; mPAWP, mean pulmonary artery wedge pressure.

It is clinically important to monitor high-risk patients and treat those who show an abnormal pulmonary arterial bed. Figure 4 shows a potential approach to exercise haemodynamics in SSc. The 6MW stress echocardiography should be considered to assess PV and LV cardiac reserve and used as a guide for definition in this high-risk cohort.

### Limitations

This is a single centre study that included a selected patient population. The data are a little limited by a large number of patients early in the study without useable echocardiographic data.^27–29^ Early in the study, many patients had incomplete echocardiographic study data (2014–2016: n=37, 2017–2018: n=12). Possibly the amount of missing data decreased as the observers gained experience. A major reason for incomplete echocardiography was missing tricuspid regurgitant velocity (65%). In this test, observers must check the tricuspid regurgitant jet using multiplane scanning and arrange the machine setting, including the velocity range to overcome this issue. The 6MW test does not require any equipment such as an ergometer, and exercise tolerance can be assessed by the 6MW distance. This method is cost-effective and simple to screen in SSc anywhere. Our outcomes had relatively soft endpoints because we focused on the very early stage of cardiac dysfunction in patients with SSc who have, in general, few hard endpoints. Previous data showed a linearity in ΔmPAP/ΔCO; however, there were limited data on this physiology.^30^ With these limitations, we believe that larger prospective multicentre studies are warranted.

## Conclusions

Impairments in both the PV and the LV reserve assessed by 6MW stress echocardiography were associated with significant increase in the risk of the composite event (heart failure hospitalisation and cardiovascular death). Thus, 6MW stress echocardiography can be applied to treat naïve patients with SSc.
