# Supplementary figures and images for: Cardiac reserve by 6-minute walk stress echocardiography in systemic sclerosis
Source: Open Heart. 2021 Feb 19;8(1):e001559. doi: 10.1136/openhrt-2020-001559 (PMC7898855; doi:10.1136/openhrt-2020-001559)

Supplemental Figure 1

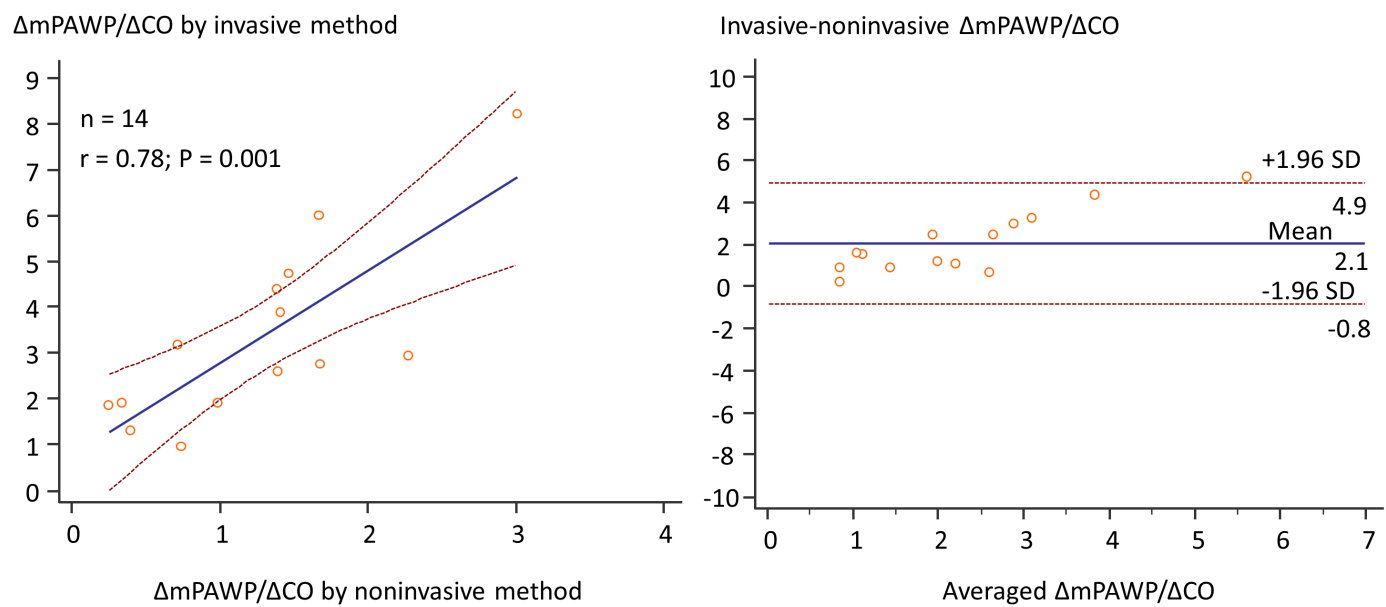

Supplement: Supplementary data [file openhrt-2020-001559supp002.pdf]
