# Supplementary material for: Cardiac reserve by 6-minute walk stress echocardiography in systemic sclerosis
Source: Open Heart. 2021 Feb 19;8(1):e001559. doi: 10.1136/openhrt-2020-001559 (PMC7898855; doi:10.1136/openhrt-2020-001559)
Supplement: Supplementary data [file openhrt-2020-001559supp003.pdf]

Supplemental Table 1. Invasive Hemodynamic Data

Variables	
Number	14
Age	62±14
Male, n	0
At rest	
HR, bpm	72±15
Systolic BP, mmHg	127±22
Diastolic BP, mmHg	68±9
Mean PAP, mmHg	20±4
Mean PAWP, mmHg	9±3
CO, L/min	6.0±2.2
At peak exercise	
HR, bpm	106±29
Systolic BP, mmHg	156±30
Diastolic BP, mmHg	78±16
Mean PAP, mmHg	42±10
Mean PAWP, mmHg	21±6
CO, L/min	10.0±2.9
ΔmPAP/ΔCO, mmHg/l/min	6.0±2.7
ΔmPAWP/ΔCO, mmHg/l/min	3.3±2.0

Abbreviations: See Table 1 and 2.
